# Overcoming the barriers to diagnosis of Morquio A syndrome

**DOI:** 10.1186/s13023-014-0192-7

**Published:** 2014-11-30

**Authors:** Kaustuv Bhattacharya, Shanti Balasubramaniam, Yew Sing Choy, Michael Fietz, Antony Fu, Dong Kyu Jin, Ok-Hwa Kim, Motomichi Kosuga, Young Hee Kwun, Anita Inwood, Hsiang-Yu Lin, Jim McGill, Nancy J Mendelsohn, Torayuki Okuyama, Hasri Samion, Adeline Tan, Akemi Tanaka, Verasak Thamkunanon, Teck-Hock Toh, Albert D Yang, Shuan-Pei Lin

**Affiliations:** Genetic Metabolic Disorders Service, The Children’s Hospital at Westmead, Hawkesbury Rd & Hainsworth St, Westmead, Sydney, NSW Australia; Metabolic Unit, Princess Margaret Children’s Hospital, Roberts Rd, Subiaco, WA 6008 Australia; Prince Court Medical Center, 39 Jalan Kia Peng, 50450 Kuala Lumpur, Wilayah Persekutuan Kuala Lumpur Malaysia; SA Pathology (at Women’s and Children’s Hospital), 72 King William Rd, North Adelaide, SA 5006 Australia; Prince of Wales Hospital, 30-32 Ngan Shing Street, Sha Tin, NT Hong Kong; Department of Pediatrics, Samsung Medical Center, Irwon-Dong, Gangnam-Gu Seoul, South Korea; Department of Radiology, Woorisoa Children’s Hospital, Guro-gu, Seoul, 152-862 South Korea; Department of Laboratory Medicine, National Center for Child Health and Development, 2-10-1 Okura, Setagaya-ku, Tokyo 157-8535 Japan; Department of Metabolic Medicine, Royal Children’s Hospital, Herston, QLD 4006 Australia; Department of Pediatrics, Mackay Memorial Hospital, No. 92, Sec. 2, Zhongshan N. Road, Taipei City, 10449 Taiwan; Children’s Hospitals & Clinics of Minnesota, 2525 Chicago Ave, Minneapolis, MN USA; National Heart Institute, 145 Jalan Tun Razak, 50586 Kuala Lumpur, Wilayah Persekutuan Kuala Lumpur Malaysia; Ipoh Specialist Hospital, Ipoh, Perak Malaysia; Osaka City University Graduate School of Medicine, 1-4-3 Asahimachi, Abeno-ku Osaka, 545-8585 Japan; Queen Sirikit National Institute of Child Health, 420/8, Ratchawithi Road, Thung Phaya Thai, Khet Ratchathewi, Bangkok 10400 Thailand; Department of Paediatrics and Clinical Research Centre, Sibu Hospital, KM 5 1/2, Jalan Ulu Oya, 96000 Sibu, Sarawak Malaysia; Changhua Christian Hospital, 135 Nanxiao St, Changhua City, Changhua County 526 Taiwan

**Keywords:** Mucopolysaccharidosis, Morquio A syndrome, Diagnosis, Skeletal dysplasia, Asia Pacific

## Abstract

**Background:**

Morquio A syndrome is an autosomal recessive lysosomal storage disease often resulting in life-threatening complications. Early recognition and proficient diagnosis is imperative to facilitate prompt treatment and prevention of clinical complications.

**Methods:**

Experts in Asia Pacific reviewed medical records focusing on presenting signs and symptoms leading to a diagnosis of Morquio A syndrome.

**Results:**

Eighteen patients (77% female) had a mean (median; min, max) age of 77.1 (42.0; 0.0, 540.0) months at symptom onset, 78.9 (42.0; 4.5, 540.0) months at presentation and 113.8 (60.0; 7.0, 540.0) months at diagnosis. Orthopedic surgeons and pediatricians were most frequently consulted pre-diagnosis while clinical geneticists/metabolic specialists most frequently made the diagnosis. Delayed diagnoses were due to atypical symptoms for 5 patients (28%), while 4 patients (22%) experienced each of subtle symptoms, symptoms commonly associated with other diseases, or false-negative urine glycosaminoglycan analysis. Two patients (11%) each experienced overgrowth within the first year of life. Two patients with Morquio A syndrome (11%) were diagnosed with craniosynostosis and 1 (6%) for each of Legg-Calvé-Perthes disease, Leri-Weill syndrome, and pseudoachondroplasia. Early radiographic features of Morquio A syndrome led to more efficient diagnosis.

**Conclusions:**

Increased awareness of clinical symptomology overlapping with Morquio A syndrome is essential. Clinicians encountering patients with certain skeletal dysplasia should consider Morquio A syndrome in their differential diagnosis. Atypical or subtle symptoms should not eliminate Morquio A syndrome from the differential diagnosis, especially for patients who may have non-classical phenotype of Morquio A syndrome.

## Background

Morquio syndrome (mucopolysaccharidosis IV, MPS IV) is a rare autosomal recessive lysosomal storage disease that includes Morquio A and Morquio B [1]. Morquio A is characterized by a deficiency of the enzyme N-acetylgalactosamine-6-sulfatase (GALNS) while Morquio B is a distinct disease characterized by a deficiency of beta-galactosidase (GLB1). The reduced GALNS activity of Morquio A results in impaired catabolism of the glycosaminoglycans (GAGs) keratan sulfate (KS) and chondroitin-6-sulfate (CS) in various tissues and organs [2,3], and leads to the multi-systemic manifestations of the disease. Common initial skeletal symptoms of Morquio A include short stature [4,5] and dysostosis multiplex [6] with abnormal gait, genu valgum, pectus carinatum and kyphoscoliosis [7,8]. Kyphosis and pectus carinatum are often present before the first year with gibbus observable before the age of two years in patients with a classical phenotype [9]. In contrast to the joint stiffness observed in other MPS subtypes, joints in Morquio A are typically hypermobile secondary to ligamentous laxity [10]. Mobility is frequently impaired for Morquio A patients, significantly impacting patient-reported quality of life (QOL) outcomes [5]. The cardiovascular, respiratory, auditory, and visual systems are also frequently affected in patients with Morquio A [8,11,12]. Although intelligence and the central nervous system remain primarily unaffected, patients with Morquio A are at significant risk for atlantoaxial subluxation and spinal cord compression [8,11,13,14]. Early surgical intervention is often required to mitigate these risks and hence, accurate early identification is imperative [13]. However, with over 220 mutations identified in the gene encoding GALNS [15], phenotypic heterogeneity makes Morquio A difficult to identify early in the disease progression and, for those with a non-classical form of the disease, erroneous diagnostic labels may lead to an inaccurate or delayed diagnosis [8-13].

Morquio A syndrome is a progressive disease that may ultimately result in life-threatening complications for all patients, not just those with a classical phenotype [13]. As enzyme replacement therapy (ERT) is now available for patients with Morquio A syndrome, early recognition of the clinical signs and efficient diagnosis for all patients with Morquio A becomes even more imperative to facilitate prompt treatment and prevention of the devastating clinical sequelae of the disease [11,13,14]. To this end, a group of experts experienced in diagnosing or managing individuals with Morquio A from major institutions in the Asia Pacific region recently gathered to discuss the diagnostic challenges they have encountered, determine common and unique clinical manifestations within their cohort of patients, and elucidate any potential solutions to those challenges encountered during the diagnostic process of Morquio A.

## Methods

A group of multidisciplinary healthcare professionals (HCPs), including geneticists, metabolic specialists, pediatricians, radiologists, orthopedic surgeons, and cardiologists, currently or previously involved in diagnosing or managing individuals with MPS from the Asia Pacific region gathered for a two-day workshop in Hong Kong in September, 2013 to discuss the referral and diagnostic process for Morquio A at their institutions. Prior to the meeting, each HCP reviewed the medical history of some cases of Morquio A syndrome at their institution. The diagnosis was considered challenging if there were subtle or atypical presenting symptoms, a misdiagnosis prior to confirmed diagnosis of Morquio A, delays to diagnosis, or normal or only marginally elevated urine GAG (uGAG) results. Cases that were identified as having encountered challenges to the diagnostic process were included in the analysis. Approval from Institutional Review Boards was not required as no experimentation was undertaken. Written patient consent was obtained from all patients whose case or images were presented.

Medical records were reviewed, with a focus on presenting signs and symptoms that led to the diagnosis of Morquio A. Clinical and laboratory information such as uGAG analysis, phenotype, disease progression, complications, and disease burden were included for analysis. Data was amalgamated and analyzed to identify patterns in symptomatology. The diagnostic processes, referrals, and time to diagnosis were analyzed to determine the effectiveness of certain processes. Awareness of Morquio A among certain specialists, and symptomatology, which may be associated with a delay in diagnosis or misdiagnosis, were also analyzed. All challenges encountered were assessed by the authors, who then provided recommendations on how to address similar issues in the diagnostic process of Morquio A.

## Results

The details of diagnostic processes for 18 patients (77% female) with Morquio A syndrome presented at the 16 participating institutions in the Asia Pacific region were available for analysis. The mean (median; min, max) age at symptom onset was 77.1 (42.0; 0.0, 540.0) months for all patients and 53.0 (42.0; 3.0, 192.0) months when removing the outliers. Outliers included one infant who presented at birth with classical symptoms of Morquio A and was diagnosed within the first year of life and one patient with non-classical phenotype who was not diagnosed until his mid-forties. Although patients presented to physicians at a mean age (median; min, max) of 78.9 (42.0; 4.5, 540.0) months for all patients and 54.8 (42.0; 12.0, 192.0) months for patients when removing the outliers, the mean age (median; min, max) at diagnosis was 113.8 (60.0; 7.0, 540.0) months for all patients and 93.9 (60.0; 14.0, 324.0) months when outliers are removed. This delay in diagnosis between age at presentation and age at diagnosis in our cohort was attributed to the atypical symptoms experienced by 5 patients (28%), 4 patients (22%) experiencing subtle symptoms, and 4 (22%) patients with symptoms most commonly associated with other diseases. Four patients (22%) also had uGAG levels that did not surpass >5 mg/mmol creatinine to indicate a positive screening result for Morquio A, but were later diagnosed by enzyme measurement or molecular analysis with known pathological mutations being identified. One of these patients, who had been diagnosed through radiologic evidence of the disease, had normal urine electrophoresis even though it was repeated three times and in two different laboratories (Table 1).

**Table 1:** **Demographics and the pre-diagnostic data from the medical chart review of patients diagnosed with Morquio A syndrome**

**Information from medical charts**	
**Age at symptom onset mean (median; min, max) months**	
All n =18	77.1 (42.0; 0, 540)
Without outlier n =16	53.0 (42.0; 3.0, 192.0)
**Age at first consultation mean (median; min, max) months**	
All n =18	78.9 (42.0; 4.5, 540.0)
Without outlier n =16	54.8 (42.0; 12.0, 192.0)
**Age at diagnosis mean (median; min, max) months**	
All n =18	113.8 (60.0; 7.0, 540.0)
Without outlier n =16	93.9 (60.0; 14.0, 324.0)
**Diagnoses identified for study inclusion**	**N (%)**
Australia	7 (39%)
South Korea	4 (22%)
Taiwan	3 (17%)
Japan	2 (11%)
Thailand	1 (6%)
Hong Kong	1 (6%)
**Gender**	**N (%)**
Female	14 (78%)
Male	4 (22%)
**Pre-diagnostic key symptoms and findings**	**N (%)**
Short stature	13 (72%)
Pectus carinatum	9 (50%)
Genu valgum	8 (44%)
Spinal abnormalities	7 (39%)
Gibbus/kyphosis	5 (28%)
Scoliosis	3 (17%)
Atlantoaxial instability	3 (17%)
Hip dysplasia	4 (22%)
Impacted joint range of motion	2 (11%)
Joint pain	2 (11%)
Advanced bone age	2 (11%)
Dermal melanocytosis	2 (11%)
Spinal cord compression	2 (11%)
Overgrowth within one year of age (“big baby”)	2 (11%)
• Other skeletal abnormalities experienced by patients included arachnodactyly shortened distal phalanges, dysplastic and fragmented proximal femoral epiphysis, flattening of femoral heads, acetabular irregularities, joint space narrowing	
• Other general symptoms experienced by patients included upper respiratory infections, hernia repair, unsettled behavior	
**Impediments to diagnosis**	**N (%)**
Atypical symptoms	5 (28%)
Subtle symptoms	4 (22%)
Symptoms associated with other diseases	4 (22%)
Marginally elevated or normal uGAG levels	4 (22%)
Delayed clinical suspicion	2 (11%)
Other radiologic differentials (pseudoachondroplasia)	1 (6%)
**Misdiagnosis and other clinical diagnoses prior to Morquio A diagnosis**	**N (%)**
Spondyloepiphyseal Dysplasias	5 (28%)
Gibbus/kyphosis	5 (28%)
Scoliosis	3 (17%)
Craniosynostosis	2 (11%)
Advanced bone/dental age	2 (11%)
Overgrowth	2 (11%)
Genu valgum	2 (11%)
Corneal opacity	2 (11%)
Cardiac conduction abnormality	2 (11%)
Legg-Calvé-Perthes Disease	1 (6%)
Leri-Weill syndrome	1 (6%)
Marfan syndrome	1 (6%)
Sotos syndrome	1 (6%)
Pseudoachondroplasia	1 (6%)
Truncal hypotonia	1 (6%)
Hypertolorism	1 (6%)
Splenomegaly	1 (6%)
Growth hormone deficiency	1 (6%)
Torticollis	1 (6%)
Sleep obstruction (snoring)	1 (6%)
Cervical lymphadenopathy	1 (6%)
Apnea/hypopnoea	1 (6%)
Rheumatoid arthritis	1 (6%)
Harrison sulcus	1 (6%)
Lumbar lordosis	1 (6%)
Reversed Madelung deformity	1 (6%)
Autism	1 (6%)
**Specialist responsible for Morquio A diagnosis**	**N (%)**
Geneticist	10 (56%)
Radiologist	5 (28%)
Pediatrician	2 (11%)
Endocrinologist	1 (6%)

Symptoms are listed separately if experienced by ≥2 patient with confirmed Morquio A syndrome. Any symptom experienced by <2 patients is combined in “Other.”

Mutational analysis was available for five patients (27.7%), all of whom were Australian. One patient, who presented at 3 months of age, was diagnosed at 12 months of age with urine KS (uKS) levels of 43.9 (reference range 5.7 – 31.4) mg/mmol creatinine and an N-acetylgalactosamine-6-sulfatase activity of 0.8 (reference range 39 – 166) pmol/min/mg protein. This patient has three heterozygous mutations: p.G247D (c.740G > A), p.W326S (c.977G > C), and p.T313A (c.937A > G). The p.G247D mutation has been associated with a severe phenotype [16]; however, neither p.W326S nor p.T313A has been reported previously. Male twins diagnosed at 4.4 years of age due to one twin presenting with cord compression at 17 months of age both had uKS levels of 37.5 (reference range <27.6) g/mol creatinine and N-acetylgalactosamine-6-sulfatase activities of 1.3 and 1.4 (reference range 39 – 166) pmol/min/mg protein. Both twins are heterozygous for both p.G301C (c.901G > T), associated with a severe phenotype [16] and p.H166-Q (c. 498 C > G), also associated with a severe phenotype [17]. Another male, who is currently six years of age with a uKS level of 14.8 (reference range <11.9) g/mol Cr and an N-acetylgalactosamine-6-sulfatase activity of 2.9 (reference range 39 – 166) pmol/min/mg protein, is homozygous for the p.M41L (c.121A > T) mutation, which is associated with a milder phenotype [18]. A female patient with a uKS level of 13.4 (reference range <11.9) mg/mmol creatinine and an N-acetylgalactosamine-6-sulfatase activity of 1.4 (reference range 39 – 166) pmol/min/mg protein is heterozygous for both the p.I113F (c.337A > T) mutation, which is associated with a severe phenotype [19], and the c.244 + 3A > G mutation, which although not previously reported, is predicted by bioinformatic analysis software to decrease the efficiency of intron splicing. This patient presented with delayed walking and fine motor skills at approximately 1.5 to 2 years of age, with diagnosis at 4.5 years of age.

**Figure 1:**
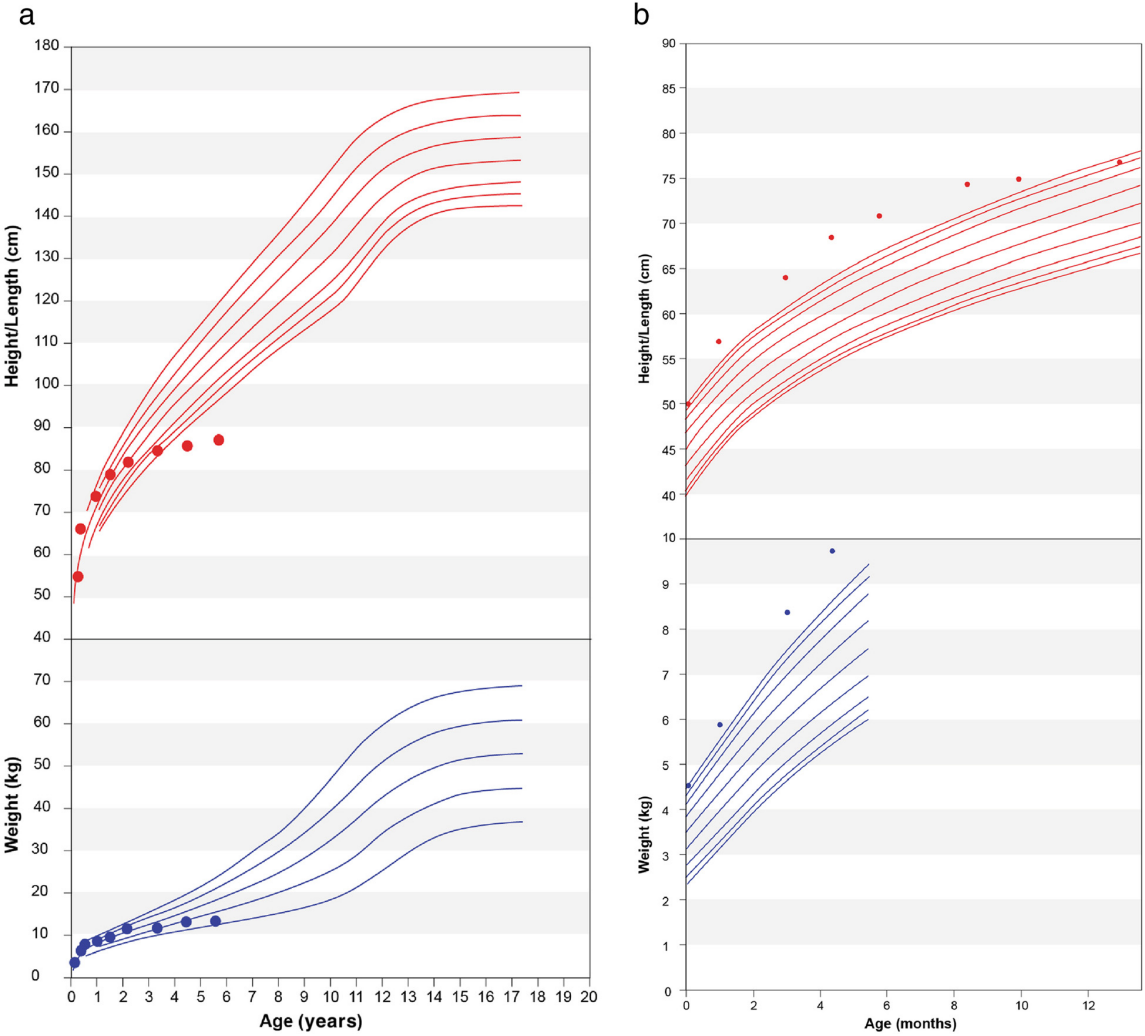
**Growth charts for a Japanese patient (a) and an Australian patient (b) diagnosed with Morquio A syndrome despite experiencing overgrowth in their early years.**

Complete information about pre-diagnostic referrals was not available, although patients were commonly referred to pediatricians, geneticists, and orthopedic surgeons. Clinical geneticists/metabolic specialists most frequently made the diagnosis of Morquio A (Table 1). Patients presented with a wide variety and spectrum of symptoms (Table 1). Symptoms not commonly associated with Morquio A that occurred in 2 (11%) patients in this cohort were overgrowth in the first year of age and extensive dermal melanocytosis (colloquially referred to as Mongolian blue spots) (Table 1). Both patients with overgrowth were diagnosed in their early years (Figure 1) including 1 patient from Australia diagnosed at 12 months based on radiographic evidence and elevated uGAG levels despite being >95^th^ percentile for length, weight and head circumference. Another patient from Japan, also classified as experiencing overgrowth within the first year of life, was diagnosed at 3.3 years with the clinical suspicion based on other physical features associated with Morquio A.

Of the 11 patients (61%) misdiagnosed in our cohort, the most common misdiagnosis was spondyloepiphyseal dysplasia (SED) and gibbus/kyphosis, both experienced by 5 patients (28%). Three (17%) patients were diagnosed with scoliosis and two patients (11%) were diagnosed with each of craniosynostosis, advanced bone/dental age, overgrowth, genu valgum, corneal opacity and conduction abnormalities (Table 1).

Patients presenting with early radiographic features typical of Morquio A were often diagnosed more efficiently than patients who did not have these common skeletal findings as initial symptoms. Six patients (33%) had initial presentation with common skeletal findings associated with Morquio A syndrome. Of these 6 patients, 5 presented with short stature, 2 with genu valgum, and 2 with other skeletal abnormalities. Five of the 6 patients were diagnosed promptly upon presentation of the skeletal symptoms.

## Discussion

The approval of ERT for Morquio A syndrome makes the early recognition and diagnosis of Morquio A syndrome even more imperative as early treatment initiation will lead to improved patient health and QOL outcomes. However, obtaining an early diagnosis can be challenging. In our cohort, mean time of diagnosis was delayed by 34.9 months or approximately 2.9 years after first consultation. When removing two outliers from the cohort, the mean age at presentation is approximately 4.5 years with diagnosis delayed until almost 8 years of age, representing a significant lapse between presentation and diagnosis. As expected, it is often those patients who are experiencing the more common symptoms associated with the more severe forms of Morquio A syndrome that are diagnosed efficiently. However, based on the limited mutational information from our cohort, even patients who present with common symptoms of Morquio A syndrome who are later identified to have mutations associated with severe phenotype may experience a delay to diagnosis, possibly due to confounding symptoms or lack of clinical suspicion. The symptoms and the diagnostic processes experienced by our cohort provide insights to commonly encountered barriers to the diagnosis of Morquio A syndrome. Knowledge of these barriers may increase the index of clinical suspicion amongst clinicians and therefore expedite the diagnostic process.

Raising clinical suspicion of Morquio A amongst clinicians frequently encountering these patients in the pre-diagnostic stage is necessary to ensure early diagnosis and treatment. However, sparse data exist on the natural history of Morquio A, especially in early childhood. Skeletal symptoms such as joint laxity, gibbus, cervical spine stenosis and/or cord compression, kyphoscoliosis, pectus carinatum, bilateral hip dysplasia, progressive genu valgum and short stature of unknown etiology should raise suspicion of Morquio A. This is especially true when in combination with some accompanying signs and symptoms of MPS such as macrocephaly, corneal clouding, loss of visual acuity, hearing impairment, recurrent middle ear infections, recurrent respiratory tract infection, valvular heart disease, rhythm abnormalities due to conduction defects, and recurrent inguinal or umbilical hernia [7,8,11,13]. However, five (28%) of the patients in our cohort experienced presenting signs and symptoms that were not previously reported as typical of Morquio A and may provide additional clues to consider during the clinical diagnosis (Table 1).

Two (11%) patients in this cohort were large for age at birth. They continued to experience overgrowth in infancy yet were diagnosed with Morquio A in childhood despite this atypical symptom. Although overgrowth has been reported in Morquio A syndrome patients [9], clinicians may associate Morquio A with reports describing growth in infancy as normal at birth but slowing in early childhood [7,13]. One patient with early overgrowth was initially assessed for congenital adrenal hyperplasia, Beckwith-Wiedemann and Sotos syndromes before diagnosis of Morquio A. Extensive dermal melanocytosis, which has previously been reported with MPS I and II [20-22], was found in several patients in this cohort (Table 1). Incomplete data from the remainder of our cohort limit conclusions regarding the frequency of overgrowth or extensive dermal melanocytosis in early childhood by patients with Morquio A and, therefore, the exact association or significance between these symptoms and Morquio A remains unclear. These findings may warrant a larger study for clarification, and also indicate that atypical symptoms should not exclude Morquio A from the differential diagnosis.

Clinical suspicion of Morquio A is typically based on clinical presentation and interpretation of skeletal radiographs. Signs and symptoms more commonly attributed to other skeletal disorders, which may also be more familiar to most orthopedic surgeons and radiologists, may result in misdiagnoses. In our cohort, 9 patients (50%) were initially diagnosed as SED and Legg-Calvé-Perthes disease (LCPD), yet the similarities between SED, LCPD and Morquio A did not prompt the specialists to further explore other possible contributory diagnoses. This finding is aligned with published reports, in which patients with Morquio A have been misdiagnosed with SED [23,24] and LCPD [23] based on radiographic interpretation [23,24].

The bone abnormalities, short stature and neck associated with SED, and the chronic pain and progressive deterioration of the femoral heads seen with LCPD are also commonly experienced by individuals with Morquio A. However, manifestations commonly associated with and unique to typical Morquio A such as genu valgum and joint laxity [13] may quickly distinguish these patients from those with SED or LCPD.

**Figure 2:**
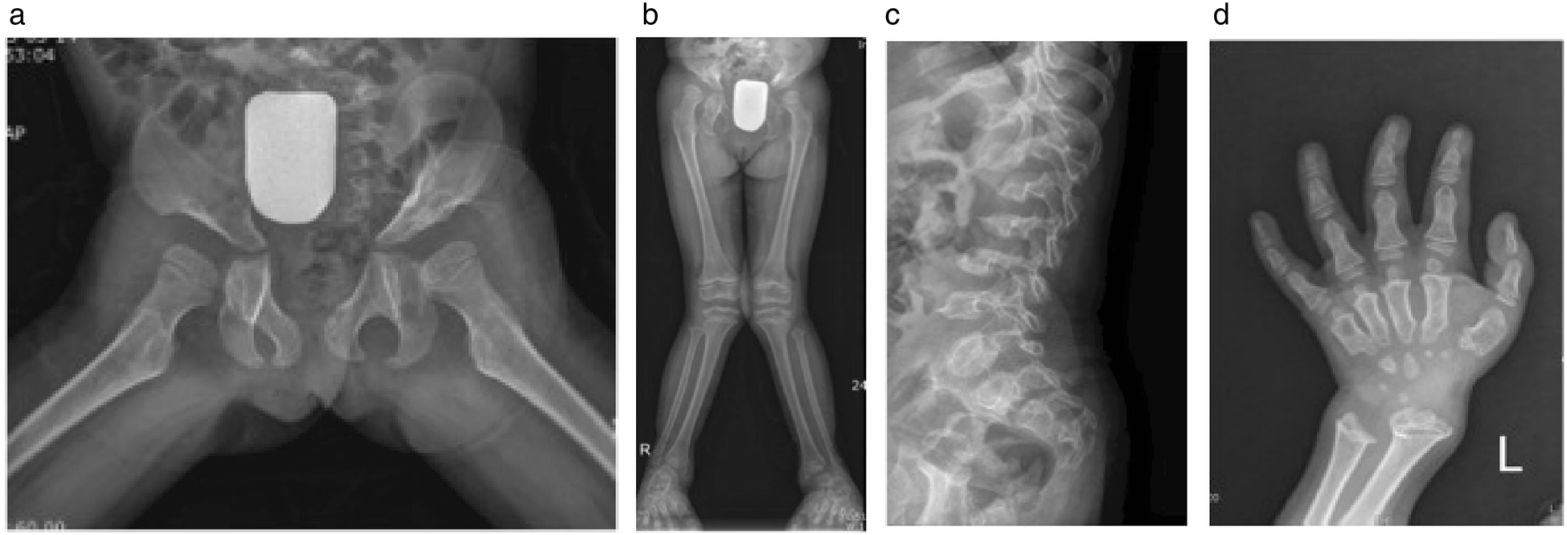
**When the mutation study for a six-year-old girl with genu valgum and short stature revealed no** ***COMP*** **gene mutation indicating pseudoachondroplasia, the patient was referred to an orthopedic clinic with resulting skeletal survey suggestive of dysosotosis multiplex and Morquio A subsequently confirmed through enzyme analysis (a-d).** **a)** The frog leg position of pelvis shows flared iliac wings with deficient ossification of the supra-acetabular portion and tapering ilium distally; ischium and pubic bones are thickened for her age; femoral capital epiphyses show slight flattening but not significant dysplasia. **b)** Lower extremity radiograph reveals coxa valga and genu valga; mild metaphyseal flaring is present in the distal femora but the distal femoral epiphyses are normal; the proximal tibial epiphyses show mild flattening. **c)** Lateral spine of the thoracolumbar vertebrae shows remarkable platyspondyly with middle beaking. **d)** Metacarpals are short with proximal conical shape that centrally converges; phalanges are short but the diaphyseal constriction is preserved; carpal bones are small and irregular; distal ulnar is short and growth plate is inclined toward the radius; radial metaphysis is wide and irregular.

**Figure 3:**
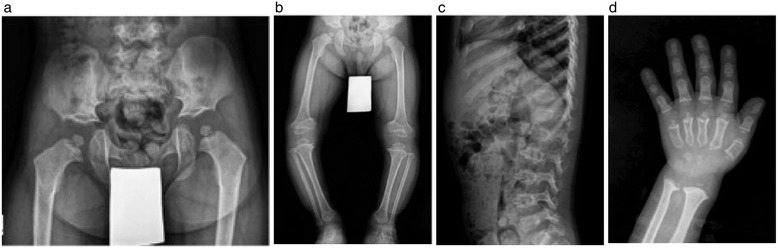
**Pseudoachondroplasia in a four-year-old boy, who presented with genu vara and short stature (a-d).** **a)** Pelvis shows small and round capital femoral epiphyses with an underdeveloped and irregularly shaped lower portion of the ilium that results in horizontal appearance of the acetabular roofs. **b)** The lower extremity radiograph shows genu vara with wide and strikingly irregular distal femoral metaphases; epiphyses at the knees that are dysplastic and triangular in shape in the femoral ends that partly invaginated into the cupping of the femoral metaphases; dysplastic metaphyseal change is also noted in the distal tibiae. **c)** The spine radiograph shows mild platyspondyly and considerable irregularity of the upper and lower endplates of the vertebral bodies; mid-central tongue in pseudoachondroplasia associated with apophyseal dysplasia of the vertebral bodies along the upper and lower endplates, resulting in biconvex configuration. **d)** Short metacarpals with metaphyseal cupping and irregularity and small, round epiphyses; short and stubby phalangeal bones with mild metaphyseal cupping; delayed ossification of the small and irregular carpal bones with apparent metaphyseal widening and irregularity at the distal radius and ulna.

Diagnosis of SED based solely on the presence of scoliosis, kyphosis, and/or a single superior notched vertebra without the presence of either platyspondyly or superior/inferior humping of vertebral bodies is not consistent with any known form of SED [24] and consideration of an alternative diagnosis such as Morquio A is prudent. Although patients of non-classical phenotype may not have the typical, pronounced manifestations that would lead to a clinical suspicion of a MPS disease [23-25], awareness of the unique and distinguishing features of orthopedic diseases may contribute to earlier diagnosis of Morquio A. There are subtle radiographic differences between Morquio A (Figure 2a-d) and other skeletal dysplasia, such as pseudoachondroplasia (Figure 3a-d) and SED (image not shown), which could help clinicians distinguish between these diagnoses. The pelvis and knee changes observed in patients with Morquio A (Figures 2a and 2b) differ from those with pseudoachondroplasia (Figures 3a and 3b). The spine of a patient with pseudoachondroplasia (Figure 3c) shows mild platyspondyly and considerable irregularity of the upper and lower endplates of the vertebral bodies. The anterior protrusion of the central aspects of the vertebral bodies may mimic to the middle beaking in Morquio A (Figure 2c), but this mid-central tongue in pseudoachondroplasia is associated with apophyseal dysplasia of the vertebral bodies along the upper and lower endplates, resulting in biconvex configuration (Figure 3c).

**Figure 4:**
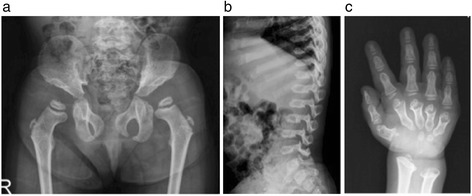
**Radiographic evidence of Morquio A syndrome in a three-year-old female Morquio A patient (a-c).** The pelvic radiograph **(a)** shows steep acetabulum with tapering ilium distally. Capital femoral epiphyseal dysplasia is noted. The lateral image of the thoracolumbar spine **(b)** shows uniform platyspondly with mild middle beaking. The image of the hand **(c)** shows strikingly short metacarpals with proximal coning and convergence toward centrally and phalanges that retain diaphyseal constriction. The distal radius and ulna inclined on their planes towards each other.

**Figure 5:**
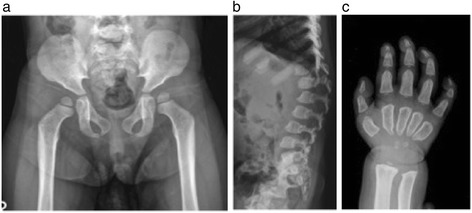
**Radiographs of a two-year-old female patient diagnosed with Hurler syndrome (MPS IH) (a-c).** **a)** Flaring iliac wings with distal tapering; capital femoral epiphyses are not dysplastic. **b)** Hypoplastic L2 vertebral body with dorsal gibbus maximized at this point; inferior beaking (hook shape) throughout the lumbar vertebral bodies; posterior scalloping of the vertebral bodies in the lumbar spine. **c)** Short metacarpals with tapering and converging of proximal ends; diaphyseal widening through the metacarpals and phalanges; distal radius and ulnar are inclined on their planes towards each other.

Subtle radiographic distinctions also exist between Morquio A and other MPS subtypes (Figures 4a-c, 5a-c). While the pelvis of a patient with Morquio A would have a steep acetabulum with tapering ilium distally with capital femoral epiphyseal dysplasia (Figure 4a), a patient of approximately the same age with Hurler syndrome (MPS IH) would have similar appearances but without dysplastic capital femoral epiphyses (Figure 5a). Patients with Hurler syndrome commonly have a hypoplastic L1 or L2 vertebral body, dorsal gibbus, and posterior scalloping of the vertebral bodies of the lumbar spine, yet do not have short vertebral bodies (Figure 5b), in contrast to a patient with Morquio A whose lateral thoracolumbar spine shows uniform platyspondyly with mild beaking (Figure 4b). While the phalanges of a patient with Morquio A retain its diaphyseal constriction (Figure 4c), there is diaphyseal widening through the phalanges and metacarpals for patients with Hurler syndrome (Figure 5c). As well, the joint hypermobility commonly associated with Morquio syndrome in the early stages may distinguish patients with Morquio A patients when compared with the fixed or constricted joint seen in other MPS disorders. Both lead to functional impairment but in the former, adaptations to hand function, in particular, address passive hypermobility at the wrist and decreased strength. In the latter the interventions increase range of movement [26]. This distinction is important in order to correctly manage the identified problems. In Morquio, the pathophysiology of this difference could be related to impaired chondrocyte function leading to defective growth plate architecture. Whilst some chondrocyte and collagen dysfunction has been noted in post-mortem studies of adults, conclusions cannot be drawn as no systematic prospective study has yet taken place of the children as they acquire these difficulties [27,28].

Laboratory challenges may further complicate the diagnostic process for Morquio syndrome, as with all MPS disorders. However, the laboratory testing for Morquio A is more challenging than in the other MPS disorders and may lead to false-negative results in the early onset form and especially in the non-classical phenotype [10]. Quantitative analysis of total uGAG is routinely performed as the first stage of laboratory testing when an MPS disorder is suspected. Many laboratories only perform further testing on patients with elevated uGAG levels [10]. Further, qualitative uGAG analysis, either by thin layer chromatography or electrophoresis [29,30] can also prove problematic, with KS being the most difficult GAG to separate and visualize [10]. Although using both quantitative and qualitative uGAG assessments may reduce the potential for false-negative results, these can still occur, particularly in dilute urine samples with a very low creatinine concentration. It is also recognized that patients with non-classical phenotype may not have elevated uKS levels [10], and that KS excretion in the urine decreases with age for patients with Morquio syndrome [31].

**Figure 6:**
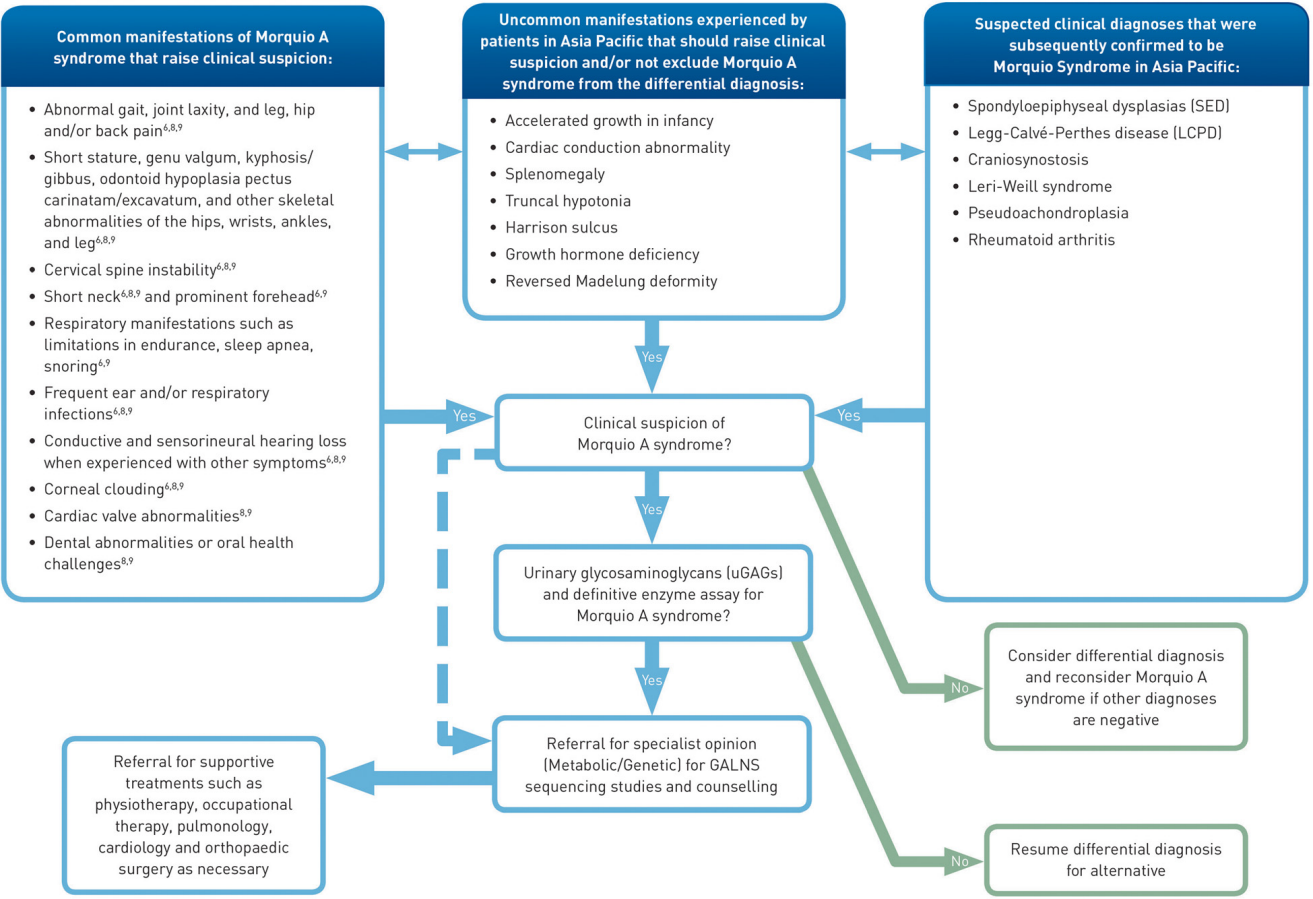
**Algorithm for raising clinical suspicion to support an efficient management process for patients with Morquio A syndrome.**

With such a large percentage of patients experiencing delays to accurate diagnosis of Morquio A syndrome due to confounding symptoms and other clinical diagnoses in our cohort, we recommend the use of an algorithm that includes both presenting manifestations and alternate clinical diagnoses to assist clinicians who may encounter patients with Morquio A syndrome. Awareness of clinical diagnoses that are commonly made for patients who are later diagnosed with Morquio A syndrome is imperative to streamline the diagnostic process for these patients. Based on our cohort, this is especially important when a patient is clinically diagnosed with atypical or unclassified SED, or LCPD. Testing for these patients should include quantitative and qualitative uGAG analysis and enzymatic activity in leukocytes or fibroblasts. Due to the high potential for false-negative uGAG analysis, confirmatory testing should be explored even if the uGAG analysis is negative for Morquio A syndrome when a high degree of clinical suspicion remains (Figure 6).

Mutational analysis may assist in obtaining a conclusive diagnosis, particularly in those with a known family history of Morquio A syndrome. When using molecular testing for diagnosis, two known or probable causative mutations on separate alleles need to be identified, which is not always possible. In this cohort alone, 4 (21.0%) patients had only marginally elevated or normal uGAG results and one early onset patient had a normal electrophoresis pattern. One patient from Japan presented at 12 years with short stature and bone deformities. An orthopedic surgeon suspected SED or Morquio A based on radiographs, short stature, and pectus carinatum, despite no evidence of genu valgum or joint laxity, and referred to a pediatrician who performed the uGAG analysis which resulted in a false-negative result. Had the pediatrician not pursued enzymatic analysis due to high levels of clinical suspicion, this patient who was subsequently confirmed to have Morquio A, may have been erroneously treated for SED. Conversely, a South Korean patient in our cohort was initially diagnosed with LCPD at 11 years of age. Despite negative mutational analysis of COL2A1 for SED, the diagnosis was revised to SED at 12 years by a radiologist with a great deal of experience with Morquio A based on clinical progression of mild scoliosis, radiographic interpretation of mild platyspondyly, and the absence of the classic features of Morquio A. It was not until whole exome sequencing was carried out that the patient was diagnosed with Morquio A at 27 years of age [18]. Subsequent qualitative and quantitative uGAG analysis and retrospective enzyme analysis were consistent with the disease. If enzyme analysis had been performed earlier as a means to exclude Morquio A, costly tests such as whole exome sequencing, erroneous medical surveillance strategies, and incorrect genetic counseling of the family may have been avoided.

One critical way to improve early diagnosis of Morquio A is through continuing medical education activities such as grand rounds and case sharing or outreach programs such as consultancy with experienced specialists. One-on-one outreach programs to departments that are likely to encounter patients with Morquio A in the critical pre-diagnostic stage could increase the likelihood of developing clinical suspicion among these specialties. Developing a local or regional disease registry of individuals diagnosed for Morquio A syndrome could also further contribute to the awareness of common and atypical symptoms, improving the current understanding of the disease.

Clinicians should be made aware that cultural barriers such as denial of disease and lack of awareness amongst patients or their families could result in reluctance to seek medical attention or under-emphasis of some subtle symptoms for those with Morquio A in some Asia Pacific countries. Emphasis should be made on presenting symptoms and radiologic features associated with non-classical phenotype to ensure efficient diagnosis for all patients, not just those most severely affected who are already likely to be diagnosed in a timely manner due to the significant impact of the disease early in life. It is also important to educate healthcare professionals of the significant risks associated with Morquio A syndrome, such as atlantoaxial instability and cervical cord compression. These complications pose a serious medical concern for all patients with Morquio A syndrome, especially those not yet diagnosed, potentially exposing these patients to a significant risk of anesthetic and perioperative complications [32].

## Conclusion

Institutions throughout the Asia Pacific region encountered patients with Morquio A syndrome prior to diagnosis and are experiencing similar challenges as in other regions, as well as some cultural barriers such as reluctance to seek medical attention in certain regions of Asia Pacific. Awareness of the disease with attendant clinical features in multiple specialties through outreach programs are imperative to ensure that all signs and symptoms trigger clinical suspicion, and not just the symptoms typically associated with the classical phenotype.

### Consent

Written patient consent was received from all patients who contributed cases and images.

## Abbreviations

(MPS): Mucopolysaccharidosis
(GLB1): Beta-galactosidase
(GALNS): N-acetylgalactosamine-6-sulfatase
(GAGs): Glycosaminoglycans
(KS): Keratan sulfate
(C6S): Chondroitin-6-sulfate
(uGAG): Urinary glycosaminoglycan
(HCPs): Healthcare professionals
(SED): Spondyloepiphyseal dysplasia
(LCPD): Legg-Calvé-Perthes disease
